# Designing a Cyber-Physical System for Ambient Assisted Living: A Use-Case Analysis for Social Robot Navigation in Caregiving Centers

**DOI:** 10.3390/s20144005

**Published:** 2020-07-18

**Authors:** Luis V. Calderita, Araceli Vega, Sergio Barroso-Ramírez, Pablo Bustos, Pedro Núñez

**Affiliations:** Laboratory of Robotics and Artificial Vision, Department of Computer and Communication Technology, University of Extremadura, 10003 Cáceres, Spain; avegamag@alumnos.unex.es (A.V.); sbarmirez@unex.es (S.B.-R.); pbustos@unex.es (P.B.); pnuntru@unex.es (P.N.)

**Keywords:** social robotics, social navigation, ambient assisted living, cyber-physical system

## Abstract

The advances of the Internet of Things, robotics, and Artificial Intelligence, to give just a few examples, allow us to imagine promising results in the development of smart buildings in the near future. In the particular case of elderly care, there are new solutions that integrate systems that monitor variables associated with the health of each user or systems that facilitate physical or cognitive rehabilitation. In all these solutions, it is clear that these new environments, usually called Ambient Assisted Living (AAL), configure a Cyber-Physical System (CPS) that connects information from the physical world to the cyber-world with the primary objective of adding more intelligence to these environments. This article presents a CPS-AAL for caregiving centers, with the main novelty that includes a Socially Assistive Robot (SAR). The CPS-AAL presented in this work uses a digital twin world with the information acquired by all devices. The basis of this digital twin world is the CORTEX cognitive architecture, a set of software agents interacting through a Deep State Representation (DSR) that stored the shared information between them. The proposal is evaluated in a simulated environment with two use cases requiring interaction between the sensors and the SAR in a simulated caregiving center.

## 1. Introduction

The development of the so-called Cyber-Physical Systems (CPS) has become very popular in the last decade. They are the basis of the Smart-Cities and Communities, and their benefit for modern societies will be a reality in the coming years. The main objective of a Cyber-Physical System is to improve the performance of a real Internet of Things (IoT) system, connecting the physical devices that acquire measurements and knowledge of the environment, with software components and agents that allow addressing actions to a specific goal. Technologies used in Cyber-Physical Systems, such as Cloud Computing, Big Data, Artificial Intelligence, or Robotics, are evolving quickly in recent years, which augurs the take-off of these systems for multiple purposes.

One of these main objectives that focus the attention of modern societies is how to deal with the aging of the population. This demographic change is a real and complex problem that governments must address through policies that ensure the improvement of the quality of life of the elderly. Numerous studies confirm this aging of the population, such as those proposed by the United Nations Foundation which states that one in six people in the world will be over 65 (16%) [1], or the Eurostat report which predicts that the relative share of the total population will also gradually increase and is projected to reach 28.5% in 2050 [2]. Among the objectives, priorities are those of preserving the health, safety, and independence of older people.

Therefore, older people must have an active mental, physical, and emotional state that allows them to increase their independence and quality of life in their own homes or in nursing homes [1]. It will be necessary to transform these nursing homes and caregiving centers to provide elder-centered services that increase this autonomy and independence. CPSs are a crucial element in achieving these goals, since they provide abilities to observe the user and environment conditions in a non-invasive way and to take specific actions depending on them. A growing number of authors propose CPS in healthcare (an interesting review is found in [3]), which demonstrates the importance of this topic in the scientific community. In all of them, CPSs are composed of a set of sensors that acquire information from the user and the environment to generate remote responses from the own system or from the caregivers. Motivated by these pioneering initiatives, this article describes the design of a new CPS for elderly care, where besides the physical devices deployed in the environment, a Socially Assistive Robot (SAR) is integrated into the architecture.

Socially Assistive Robots are robots designed for social interaction with humans and carry out their activity in everyday environments. These SARs provide, on the one hand, an interface for the elderly to access digital technology, while on the other hand, the SARs’ company can help to increase their quality of life [4]. However, their skills are limited to the robot’s perception system, i.e., their specific actions depend on the SAR’s sensor array (e.g., a single camera, a microphone...). To avoid these real limitations, the integration of SARs in smart environments is a novel strategy. A social robot-integrated smart environment builds a digital ecosystem that can, among other functions, personalized treatment, long-term monitoring, communication, and therapy. These technologies for active aging are included under the term Ambient Assisted Living (AAL) [5] and represent the research line that motivates the presented work.

Specifically, achieving the integration of many different physical devices, each with distinct interfaces, specific communication technologies, or particular driver software, is a difficult task. The main research objective is to verify the viability of a new CPS that integrates the information of a socially assistive robot (SAR) as another device of the physical world, with the ability to move and that, besides perceiving, is capable of acting in the environment and interacting with people. Furthermore, this CPS is built based on a digital twin world from all the information acquired by the physical world, and that offers the tools to learn and make decisions that successfully carry out the specific actions by the robot or by the system itself.

Although technology will never replace a professional or a family member when is caring for an older person, the development of a CPS for elderly care can provide them with a more independent and better quality of life. The novel framework described in this article, named CPS-AAL, currently facilitates the interaction over communication networks between the different agents of the CPS-AAL, which are located on multiple computational platforms. Moreover, the proposed CPS-AAL includes human and a robot as integral parts of the system, which is also a novelty in similar approaches. In addition, it is scalable and modular, both in the physical world and in the cyber-world, facilitating its adaptation to possible changes in the infrastructure (improvement of perception systems, improvements in specific algorithms) or adding new equipment or functionalities.

The CPS-AAL will be evaluated in a specific application, the social navigation of a robot in an environment with humans. This use case is of primary importance for most applications where robots interact with people. Social robot navigation is a complex task, but necessary for other essential robotics skills. To carry out this objective, the CPS-AAL must be able to detect people and objects and observe possible interactions between them and plan paths. Moreover, taking into account that people and objects in the environment can change their positions and the system must respond appropriately.

This paper is organized as follows. Section 2 presents a general overview and related background of CPSs in elderly care. In Section 3 the general overview of the CPS-AAL proposed in this research is presented, which revolves around the different IoT infrastructures. Section 4 focuses on the specific use case, describing the involved subsystems, including the experimental results and the main discussion on the lessons learned from this experience. Finally, Section 5 presents the main conclusions of this work as well as an outlook on future research lines.

## 2. Overview of Cyber-Physical Systems in Caregiving Environments

The evolution of CPSs is an objective fact, involving more and more areas of daily life. Revolution 4.0, as it has been called in the scientific literature, has been made possible by that step forward in engineering and technology. This evolution has been possible thanks to the development and implantation of CPSs in different areas of interest in modern societies [6]. Industry 4.0, closely related to the future of manufacturing, depends directly on key issues related to CPS and IoT technologies [7]. Although traditionally industry is the one that has been able to adapt more and better to the evolution of IoT technologies, there are other applications where the deployment of CPS is being explored. In fact, CPSs are also an integral part of Agriculture 4.0, Medicine 4.0, or Education 4.0 [7,8]. In all of them, the advances in CPSs are an essential goal in the building of developed societies. In this section, a general overview of CPSs and its main characteristics are provided.

### Cyber-Physical Systems and Healthcare Initiatives

Cyber-Physical Systems can provide more intelligence to social life by integrating physical devices with cyber agents to form a smart system that responds to dynamic changes in real-world scenarios. CPS is formally described in Lee et al.’s work as an integration of computation with physical processes whose behavior is defined by both cyber and physical parts of the system [9].

A crucial feature of a CPS is the interbreeding of IoT technologies, Big Data and Cloud Computing. Different research lines address this issue, which involves the definition of CPS architectures, such as those described in [10,11,12]. In [10], authors propose a 5-level CPS architecture (5C) for developing and deploying a CPS for manufacturing applications, from the initial data acquisition to the final value creation. This 5C architecture defines the integration of 5 inherent components, namely connection, conversion, cyber, cognition, and configuration, where each level has described its main functions and attributes. Nie et al. [11] present in detail a three-level architecture for precision agriculture, the physical layer, the network layer, and the decision layer. A CPS architecture for health application is proposed in [12], where authors define an architecture of three layers, namely data collection layer, data management layer, and application service layer. The data collection layer is used in the 3-level architecture for the integration of public medical resources and personal health devices, while the same CPS has a cloud-enabled and data-driven subsystem for multi-source healthcare data storage and analysis. New models have been proposed, such as the architectures based on the digital twin world described in [13,14]. In [13], authors establish a cyber-physical connection via decentralized digital twin models to parallel control the manufacturing system. A cloud-based digital twin architecture reference model is also defined in [14], where its digital twin model, its cyber-world model, is composed as a set of finite state machines. Each one of these architectures has been designed for a particular application, development environment, or system specifications. However, there is a consensus among most researchers that a CPS architecture should capture a variety of physical information, reliable data analysis, event detection, and security.

Although many CPS architectures have been proposed in the literature, the number of them for caregiving applications is very few. Rahman et al. [15] propose a cloud-based virtual caregiver for elderly people, which describes a necessary IoT CPS which supports in-home therapy sessions by using a set of gesture-tracking sensors and ambient intelligent IoT sensors. In [16], a simple CPS for assistive robotics technologies in the home is presented, where authors describe a case study for detecting and responding in case an older person falls at home. Haque et al.’s survey [3] reviews the use of CPSs in Healthcare, depicting the CPS scenario concerning the essential components such as application, architecture, sensing, data management, computation, communication, security, and control actuation. Concretely, in the case of the elderly, the authors summarize specific assisted applications for them that include health monitoring, both at home and caregiving center, and virtual assistance.

**Figure 1:**
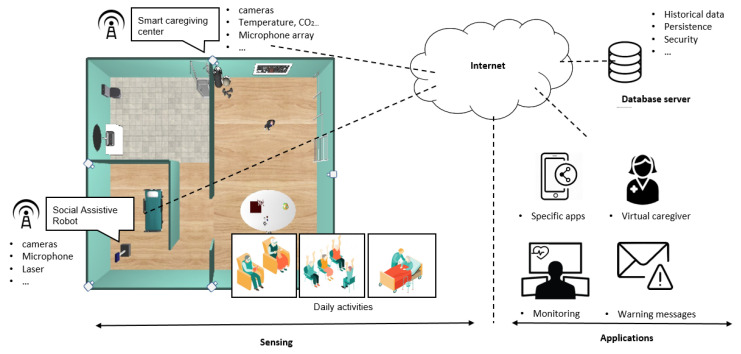
General view of a Cyber-Physical System for caregiving center.

Figure 1 depicts a CPS for caregiving environments conceived based on this literature to facilitate further discussion in subsequent sections of this paper. The possibilities of extending all the caregiving center functionalities using the advances of the IoT and the CPS are remarkable and, moreover, it is one of the main objectives of this article.

## 3. Cyber-Physical System for Caregiving Centers

**Figure 2:**
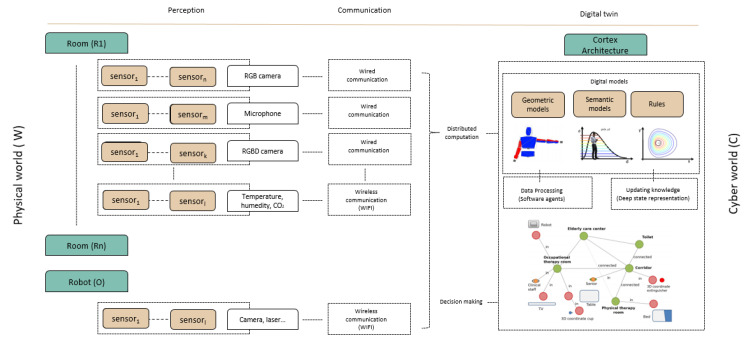
General view of the CPS-AAL proposed.

A Cyber-Physical System is a distributed, networked framework that combines data processing with the real world. A caregiving center could be understood as a typical example of CPS, where a set of sensors deployed in the environment collects real-time information (physical world) to make future decisions (cyber-world) that can be useful for assisting elderly and caregivers. The architecture of the proposed CPS-AAL is shown in Figure 2. The physical world consists of the set of devices installed in each of the rooms of the caregiving center (e.g., cameras, microphones, temperature sensors, etc.), as well as the robot itself and the sensors with which it is equipped. The data processing is done in a distributed manner, through the RoboComp framework [17]. Regarding the cyber-world, this CPS-AAL presents a digital twin world based on the CORTEX architecture described in [18], which defines a virtual shared representation of the real world. As shown in Figure 2, virtual models and rules are used as a supplement to enrich the IA algorithms. The CPS-AAL proposed in this paper forms a closed loop between the cyber and physical world based on perception, data analysis, and decision making.

The proposed CPS-AAL S is composed of several independent systems. Let W be the physical world, in charge of acquiring information from the environment, D be the system in charge of storing the data in local servers, and C be the cyber-world, the digital twin world with all the information acquired by physical devices and shared by the rest of the agents involved, which carries out data processing and decision making, then: S = (W,D,C). Next, subsections describe the proposed CPS with details.

### 3.1. Designing the Physical World

The physical world W consists of the set of all devices, sensors and actuators, deployed by the caregiving center facilities, $W_{\mathrm{AAL}}$, in addition to the socially assistive robot, $W_{\mathrm{SAR}}$. According to recent studies [19], the monitoring of users is one of the essential objectives, not only of their physical, cognitive, or emotional conditions, but also of their location in the world. Also, interacting with users is a possibility to take into account in the design of the CPS-AAL. This interaction can be direct—through auditory or visual channels and/or through human-robot interaction—or indirect, acting directly on physical devices (e.g., temperature management in rooms or alarm signal activation). This subsystem is not closed and can be extended with new devices if needed. Figure 2 shows a diagram of the physical system implemented in the caregiving center, and also it shows the physical world consists of a set of devices deployed in different rooms $R_{i}$ and the SAR.

#### 3.1.1. Ambient Assisted Living

**Figure 3:**
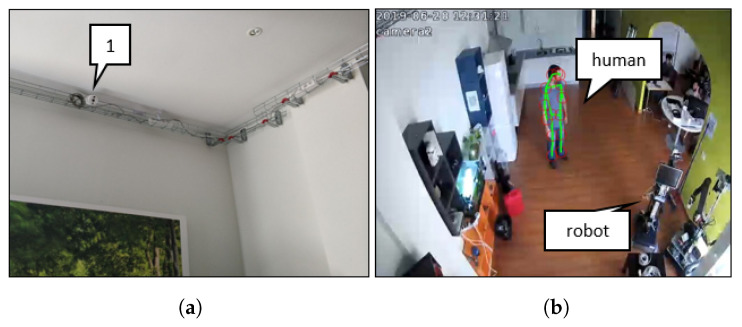
(**a**) Partial view of the physical world W with an RGB camera with wired communication; and (**b**) image capture from the camera labeled as “1” in a).

The Ambient Assisted Living must be equipped with devices that allow monitoring and providing services to different users, from the older person to the caregiver and even the robot itself. These ecosystems are equipped with physical devices capable of acquiring data from the environment, accessing data storage systems, communicating using wireless or wired, and acting on the environment. Figure 3a shows a partial view of the physical world, where an RGB camera (labeled as “1”) is highlighted, also, in Figure 3b a view from camera “1” is shown, where it is highlighted the human and the robot in the scene similarly.

In the proposed CPS-AAL the physical world $W_{\mathrm{AAL}}$ consists of a set of physical sensors and actuators, which are classified as follows: (1) ambient temperature sensors ($w_{t}\in W$); (2) relative humidity sensors ($w_{h}\in W$); (3) presence and location sensors ($w_{p}\in W$); (4) $CO_{2}$ sensors ($w_{CO_{2}}\in W$); (5) RGB/RGBD cameras ($w_{RGBD}\in W$); (6) microphones ($w_{mic}\in W$); (7) speakers ($w_{speaker}\in W$); and (8) tactile screens ($w_{tactile}\in W$). Table 1 summarizes the list of devices that have been selected for different applications.

Therefore, $W_{\mathrm{AAL}}$ can be expressed according to Equation (1):

(1) $$W_{\mathrm{AAL}}=\left(w_{t},w_{h},w_{p},w_{CO_{2}},w_{RGBD},w_{mic},w_{speaker},w_{tactile}\right)$$

In general, each device $w_{i}\in W_{\mathrm{AAL}}$ is defined by a list of components, so that $w_{i}={\left(R_{w},Y_{w},X_{w},T_{w}\right)}_{i}$ where $R_{w}$ is the component in charge of capturing the events of the real world, $Y_{w}$ the component in charge of adapting those events to the physical variables in which they are measured. $X_{w}$ is the component in charge of connecting the sensor to the LAN/WAN and providing it with data transmission capacity, and $T_{w}$ is the component in charge of sending that information to the D layer of the databases. All selected devices use either guided or wireless connection via WIFI (IEEE 802.11).

#### 3.1.2. Socially Assistive Robot

A social robot is an autonomous robot specifically designed to work in human environments. The particularity of a social robot is that it must also interact with humans following social rules (human-robot social interaction). Thus, other devices such as speakers or tactile screens are needed. Table 1 shows a collection of these devices. Following the same nomenclature, $W_{\mathrm{SAR}}$ can be expressed according to Equation (2):

(2) $$W_{\mathrm{SAR}}=\left(w_{RGBD},w_{laser},w_{sonar},w_{mic},w_{speaker},w_{tactile}\right)$$

in where each device $w_{j}\in W_{\mathrm{SAR}}$ is also defined by the same list of software components $w_{j}={\left(R_{w},Y_{w},X_{w},T_{w}\right)}_{j}$.

### 3.2. Data Storage Subsystem

To improve efficiency, the entire CPS-AAL strives to optimize the system for storing data acquired by some of the physical world’s devices $W_{\mathrm{AAL}}$. Not all readings should be stored indefinitely (e.g., robot’s position). In all those cases where it is necessary, the essential asset is data availability, persistence, scalability, and relevance. Moreover, the correct and efficient design of data storage systems is essential for the future of the CPS-AAL. With this premise, the data storage system D is made up of a time series database(TSDB).

A TSDB consists of sequences of time-stamped values and is built/optimized for this type of data in which the event’s order is relevant. This feature makes this $D_{i}$ database an ideal instrument to store the data series that are acquired in the physical layer $W_{\mathrm{AAL}}$.

*D* is composed of different time series databases, each one associated with a physical device, $D=\left\{D^{1},D^{2},\ldots ,D^{j}\right\}$, where $D^{j}$ is the database associated with the sensor $w_{j}\in W_{\mathrm{AAL}}$. $D^{j}$ is defined as a set of independent data series, where each one is defined as a tuple (timestamp, label, value). In the proposal, *D* accepts queries directly using mathematical operations and groupings in time that allow data analysis, as well as the development of artificial intelligence, to obtain information from the CPS-AAL through virtual assistants.

### 3.3. Designing the Cyber-World

**Figure 4:**
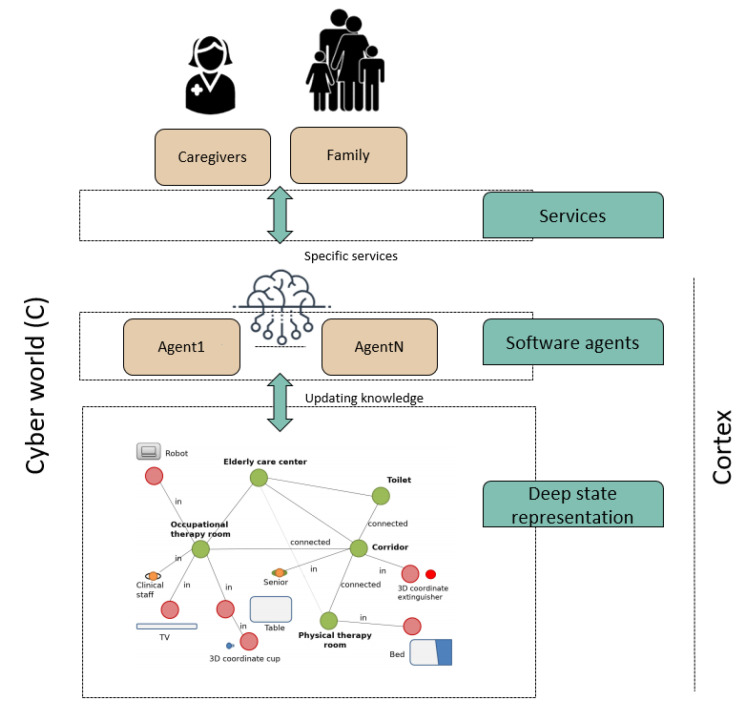
CPS-AAL architecture, which is built based on CORTEX cognitive architecture.

The main long-term objective in designing the cyber-world is to create a permanent link with the physical world to support the caregiving center’s elderly in performing specific tasks and provide caregivers with a wide range of services and applications. In the case of the use of robots in Ambient Assisted Living, where the safety of the users and the social behavior of the robot must be prioritized, it is indispensable to provide CPSs with tools that facilitate the simulation of future actions. The CPS-AAL presented in this work uses a digital twin world with all the information acquired by physical devices and shared by the rest of the agents involved, facilitating simulation for different purposes. The core of this digital twin world is the CORTEX cognitive architecture [18]. Figure 4 depicts the architecture CPS-AAL described in this work.

#### Digital Twin Model

The digital twin model C is meant, in the proposed CPS-AAL, as a virtual and computerized associated with the physical world W. The cyber-world can be used to simulate W for various purposes, exploiting a real-time synchronization of the sensed data coming from different devices and integrating them with specific models and rules. The social behavior of a robot, i.e., the robot navigating in a socially accepted way, requires the use of models based on proxemics, social rules, and even estimating future positions of the person or objects in the environment. All this justifies using a digital model as an architecture for access to historical data, sharing of information in real time, data processing, simulation of future scenarios, and action planning, among other functions.

In this work, CORTEX cognitive architecture is used as the basis of the digital twin model. CORTEX is an architecture for autonomous robots that has been successfully used in several challenging applications [20,21,22]. This architecture is based on a set of software agents interacting through a Deep State Representation (DSR) [18].

The digital twin model in this proposal is based on this DSR, defined in [22] as a multi-labeled directed graph that holds symbolic and geometric information within the same structure. This shared representation is interconnected through specific agents that incorporate models of the devices or entities required in the data processing. Furthermore, these agents are in charge of connecting with the physical world. Therefore, the digital twin model C is defined as $C=\left(G\left(N,E\right),A_{T}\right)$, being G(N,E) the multi-labeled graph composed of *N* nodes and *E* edges, and $A_{T}$ the software agents of the architecture.

**Figure 5:**
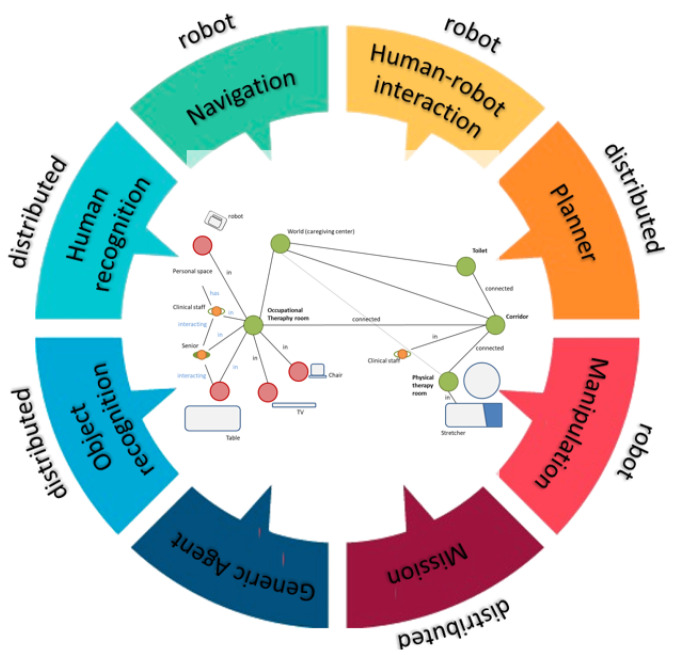
The cognitive architecture CORTEX and the multi-labeled graph DSR used in this paper as the basis of the cyber-world.

Figure 5 shows a simplified schema of the CORTEX cognitive architecture, the mind of the proposed CPS-AAL for caregiving center. The core of the architecture is this digital twin model represented like a graph with nodes (elements in the environment, such as people and objects) and edges (relationships between nodes). All agents of CORTEX work on a higher layer, and can read and modify the knowledge of the environment, i.e., the graph, which facilitates the adaptation to changes almost in real time. For example, the human-recognition agent can make use of the information from the cameras of the social robot and the cameras array in the smart environment. Achieving greater robustness in the architecture, as well as improvements in the agents’ efficiency.

To understand the digital twin model mentioned above, a more detailed description of the DSR and the CORTEX architecture is provided.

**Figure 6:**
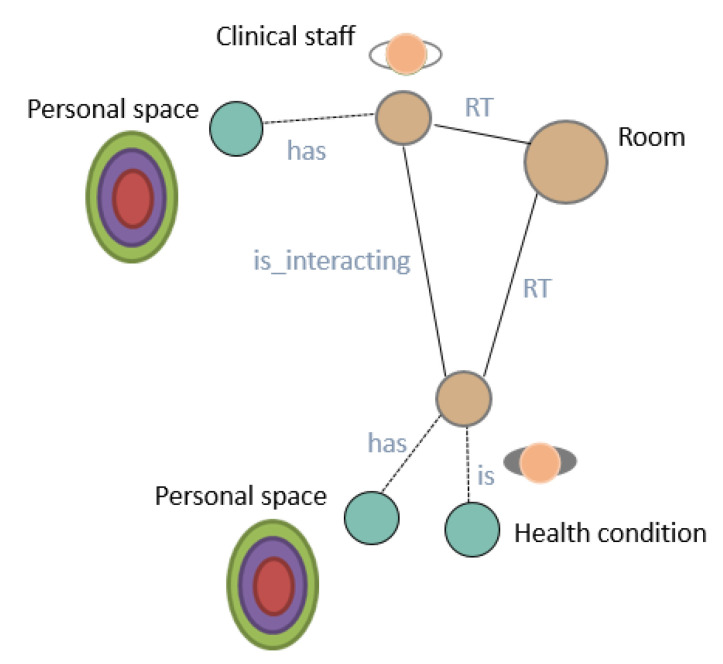
Unified representation as a multi-labeled directed graph. Edges labeled as has and is interacting denote logic predicates between nodes. Edges starting at room and end at senior and clinical staff are geometric relations and encode a rigid transformation RT between them.

- Deep State Representation.
  Figure 6 shows a simple example of the DSR for a room and a person inside. The DSR is a directed graph G(N,E), where the symbolic information states logic attributes related by predicates that, within the graph, are stored in nodes and edges, respectively. The clinical staff and senior nodes are geometrical entities, both linked to the room by rigid transformations (RT). Moreover, the senior has a particular health condition (i.e., an agent $A_{i}$ is updating this information in the graph) and both the senior and the clinical staff are interacting with each other (i.e., an agent $A_{j}$ is also annotating this situation in the graph), and each one has specific models (i.e., previous knowledge based on proxemics) of their personal spaces for decision making during social robot navigation.
  Formally, on the one hand, nodes *N* of the graph G(N,E) store information that can be symbolic, geometric, or a mix of both. Metric concepts are associated with any information associated with this node, such as temperature or humidity conditions, for example, which is directly related to the physical world W. On the other hand, edges *E* represent relationships between symbols. Two nodes $n_{i}$ and $n_{j}$ may have several kinds of relationships $e_{i,j}$, but only one of them can be geometric, which is expressed with a fixed label RT.

- CORTEX is cognitive architecture for robots and is described as a group of agents that cooperate using the DSR to achieve a particular goal. The agents at CORTEX are conceptual entities that are implemented with one or more software components. In CORTEX, the agents define classic Robotics functionalities, such as navigation, manipulation, person perception, object perception, conversation, reasoning, symbolic learning, or planning [18].
  In the proposed CPS-AAL, the network of sensors distributed in the environment enriches the DSR by enhancing the initial capabilities of the CORTEX agents. The agents also allow the implementation of actions that the CPS-AAL must carry out for elderly care: propose serious-games, notify the end of a session, or interact with the user. A brief description of the principal agents used is provided next:
  - Object recognition: The object recognition agent recognizes and estimates the position of objects in the environment. Each identified object is stored in the DSR, as a node. Its position and orientation are updated in the corresponding RT link.
  - Human recognition: Agent in charge of detecting and tracking people. This agent is in charge of detecting humans, including them in the DSR, generating the social interaction spaces, and keeping them in time. This information is used by the navigation agent, to warn the presence of humans on their route and make the necessary adjustments to try to move in a way more in line with our social norms.
  - Human-robot interaction: Agent in charge of human-robot interaction (HRI). This agent provides tools for collaboration and communication between humans and robots. The agent implements capabilities such as holding small conversations, detecting voice commands, or requesting information about unknown objects.
  - Planner (Executive): This agent is responsible for high-level planning, supervising the changes made in the DSR by the agents, and the correct execution of the plan. It integrates the AGGL planner [23] based on PDDL. The stages of the plan are completed through the collaboration of different agents. The DSR is updated and reflects the actions of each stage. This information allows the agent to use the current state of the DSR, the domain, the target, and the previous stage to update the running plan accordingly.
  - Navigation: The agent is in charge of navigating in compliance with the social rules. For this purpose, the agent is in charge of the social path-planning and SLAM. The location of the robot is updated and maintained in the DSR by this agent.

**Figure 7:**
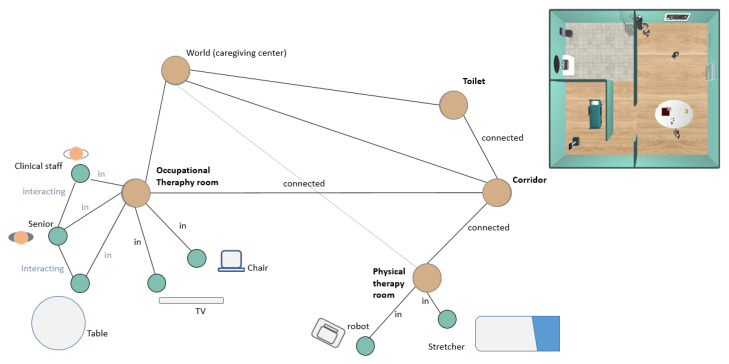
Example of the shared representation (DSR) in CORTEX. The simulated caregiving center is shown on the top right. The scenario is composed of four rooms, with objects and people within it.

Figure 7 illustrates the shared representation for the simulated caregiving center shown on the top right. In this graph, four rooms (i.e., physical and occupational therapy rooms, corridor, and toilet) are drawn as four nodes. The SAR (node robot) is in the physical therapy room, so an edge is drawn in the graph for this relationship (other types of edges are, for instance, connected, interacting, has, or on). Similarly, the rest of the digital twin model is built according to the information extracted from the physical world.

## 4. Use Case: Social Robot Navigation in Caregiving Center

Social robotic navigation is a question of massive interest in the field of autonomous robotics. Robots in scenarios with humans, such as care facilities, have to be able to behave in a socially acceptable way, i.e., it has to plan a path and navigate according to social rules, e.g., robots should avoid getting to close to people or disturbing people who are not willing to interact with them [24]. This section describes two experimental scenarios for evaluating the proposed CPS-AAL. First, a statement of the problem of socially accepted path-planning is described. Next, the use cases where the CPS-AAL is evaluated are defined, and then, the social navigation framework used in the CPS is depicted. Finally, the results are presented and discussed.

### 4.1. Problem Statement

Traditionally, when a robot navigates in real environments, most of the algorithms in the literature have considered all obstacles of similar relevance, including people. This reasoning is not valid for a social robot, who must have the ability to navigate similarly to humans. This situation implies, among other constraints, the people’s comfort level when the robot moves near them. In our opinion, two references can offer the context of the problem to the readers [25,26], and more recently [27]. This works describes the specific problem and, also, the solutions provided by other authors. An interesting approach is the definition of a social map depicted in the works [28,29], which extend the concept of the metric and semantic map to include spaces where the robot can navigate without disturbing people.

**Figure 8:**
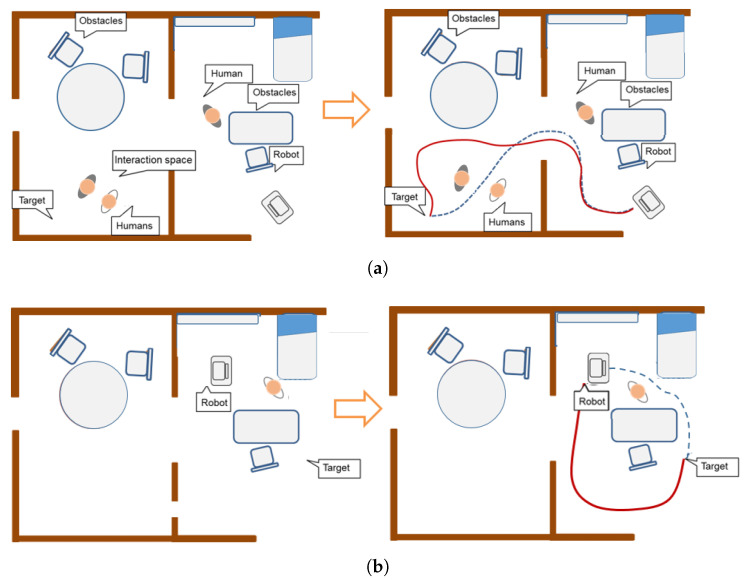
Two different everyday situations in caregiving centers: (**a**) left: a scenario where two people are interacting each other; right: red path is the only one accepted by people according to social conventions; (**b**) left: a scenario where the caregiver is interacting with the stretcher; right: red path shows the socially accepted route.

Suppose the two examples described in Figure 8, where the robot must go from the initial to the final one. The robot must avoid moving too near people or crossing between people who are interacting with each other (Figure 8a). Also, it should avoid traversing between people that are interacting with some object (Figure 8b). Consequently, the robot should path-planning according to these constraints. From this perspective, the need to model a personal space that should be included in the path-planning process to achieve acceptable behaviors for the robots during navigation seems to arise. Consequently, this article is inspired by the notion of social mapping described in [28].

This map is built in the digital twin model C from data acquired from the physical world W. To this end, the social navigation framework presented in this work requires the use of physical devices and social behavior models. In our case, these models are based on the theory of proxemics in human relationships and models of use of everyday objects [30].

To plan a socially accepted path, as concluded from the above, it is necessary a cyber-world capable of extracting information from the position of people, objects, detecting changes in those positions (tracking objects and people) and, of course, knowing the robot’s pose in the physical world at any time. It is not a simple problem and requires an architecture capable of exchanging and processing information in real time between the different agents, consistency in the data, and the use of multiple sources. It would be impossible to carry out this social navigation using only the robot’s sensors, and that is why the use of CPS-AAL has particular relevance.

### 4.2. Use-Case Definition

**Figure 9:**
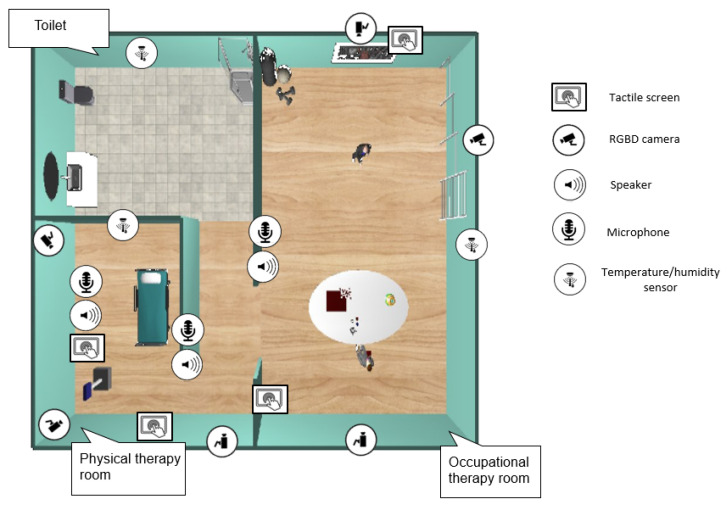
Partial view of the proposed CPS-AAL for caregiving centers.

The article presents two use cases in the scenario shown in Figure 9. It consists of a partial view of a caregiving center with two main rooms, a physical therapy room, and an occupational therapy room. Additionally, the scenario includes a corridor and a toilet. The SAR and the devices deployed in the CPS-AAL is also shown in the figure. The distribution of the sensors in the physical world has been made based on the following criteria: (1) most of the space must be visible by RGBD cameras (except for the bathroom, where there is only one camera at the entrance); (2) all rooms must have the possibility of allowing human interaction with the CPS-AAL, either through microphones/speakers or touch screens; (3) each room must have temperature-humidity and CO${}_{2}$ sensors; and (4) the number of devices installed must be the optimal one that meets the above criteria. It is also important to note that the RGBD camera network has been calibrated according to the method described in [31].

The robot has been designed to provide physical and cognitive support to aged and to help caregivers with their tasks. In particular, it communicates with users through a touch screen, speakers, and microphones for speech synthesis and recognition, respectively. On the touch screen, in addition to selecting between different services, physical and cognitive therapies are presented that the elderly can perform in collaboration with the robot. Users can communicate with the robot directly or through the array of microphones displayed on the scenario. To this end, the robot can recognize specific keywords and manage the conversation based on a dialogue manager agent.

The cyber-world, in addition to the digital twin version of the physical world, includes all the models and information necessary for the correct development of the activities in the caregiving center. Among the models used in the use cases are those related to social navigation and the construction of the social map of the robot’s environment.

**Figure 10:**
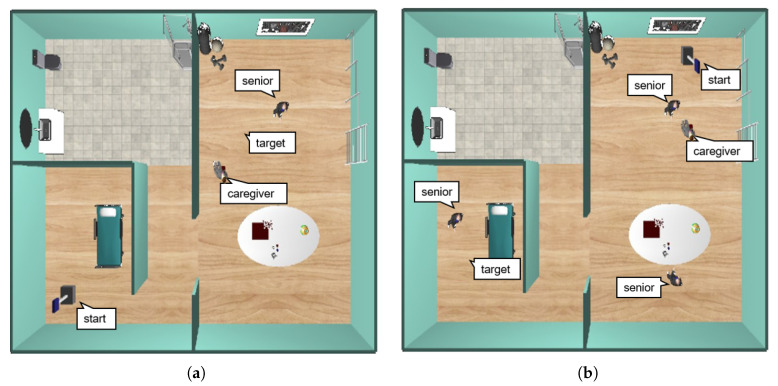
Two use cases are used in this paper to validate the proposed CPS-AAL: (**a**) The robot acts as an assistant that warns the users (older adults) that the therapy is over, and (**b**) the robot acts as a virtual physical therapist that navigates to the user and proposes a physical activity. A more detailed description of the use cases is in Table 2 and Table 3, respectively.

The first use case is described in Table 2 and Figure 10a. In this test, the robot acts as an assistant that warns the users (older adults) that the therapy is over. To performing the simulation, a senior is placed in the occupational therapy room right in front of the television (i.e., the television plays a sequence of movements that the older person is repeating). When the therapy is over, the robot navigates from its initial position to a position near the older person. Although warning the elderly can be done with any other device, such as a smartphone or smartwatch, which can be effortlessly integrated into the proposed CPS-AAL, it has been decided that the SAR will alert the old adult. The reason is to show the system’s ability to adapt the SAR’s path to social conventions since it coincides with the caregiver’s protocol: Go and warn the user that the therapy is over. This situation produces a short verbal interaction between the human and the caregiver, or the SAR in this case, which allows knowing how the therapy was developed, generating a higher degree of adherence and motivation.

The second use case is shown in Table 3 and Figure 10b. In this second test, the robot acts as a virtual physical therapist that navigates to the user and proposes a physical activity. To achieve it, the robot navigates from a starting position to the older person’s position. Once in this position, the robot begins an interaction with the senior and later presents, on its touch screen, a physical therapy that the person must imitate.

In both use cases, the entire CPS-AAL works together to achieve the same goal, starting with the agents for detecting and tracking people and objects, the human-robot interaction agents, the caregiving center management agent, which is responsible for managing, among other functions, the center’s schedule of activities, and finally the social navigation agent.

### 4.3. Social Robot Navigation Framework Based on CPS-AAL

**Figure 11:**
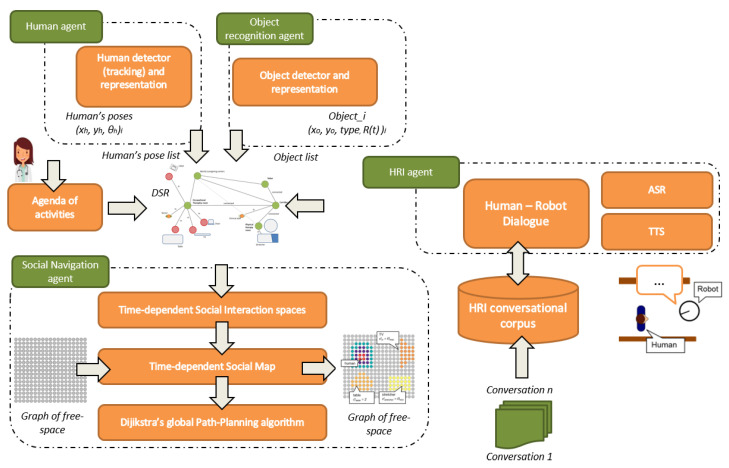
Overview of the social navigation framework within the CPS-AAL.

The framework for planning socially acceptable paths uses the CPS-AAL to acquire the information necessary to build a social map of the environment. For this reason, the CPS-AAL needs several characteristics, the first one is to detect and track people, the second one is to model their social interaction space, the third one is grouping people in a composed social space when they are interacting, and the four one is to detect and track objects, and the last one is to model their space of interaction. Furthermore, providing the robot with the ability to select specific dialogues while navigating the robot is also necessary to avoid blockages during navigation. Figure 11 shows an outline of the social navigation framework, which is more detailed in [30,32,33].

#### 4.3.1. Social Mapping Based on Interaction Spaces

Generally, people do not want to be disturbed while interacting with other people or objects. In care facilities, physical or cognitive therapies usually consist of interaction between people and between people and objects. Therefore, an SAR should detect these situations before planning its route, and at the same time, should adapt it during its navigation. The problem arises, in how to represent those regions that do not exist in reality, and that come from our social conventions. In this sense, and for the case of objects, the literature defines the concept of Space Affordances, to refer to the regions where human-object interaction takes place [34]. These spaces are called Activity spaces when people are interacting with them. In the case of people, personal spaces based on proxemics have been successfully used to represent the interaction between humans [30,35].

Building the social map of the caregiving center is one of the main objectives in this navigation framework. To achieve it, the CPS-AAL first detects people’s position in the world from the RGBD camera network. From the depth image, the agent of detection and tracking of humans provides people’s position in world coordinates. Something similar is made by the agent in charge of detecting objects in the caregiving center. Once the digital twin model has been updated, the following phases are established:

- Social mapping: people in the environment. Let $H_{n}=\left\{h_{1},h_{2}\ldots h_{n}\right\}$ be a set of *n* humans detected by the software agent, where $h_{i}=\left(x,y,\theta \right)$ is the pose of the *i*-th human in the environment. To model the personal space of each individual $h_{i}$ an asymmetric 2-D Gaussian curve $g_{i}\left(x,y\right)$ is used [30]:
  (3) $$g_{h_{i}}\left(x,y\right)=e^{-(k_{1}{\left(x-x_{i}\right)}^{2}+k_{2}\left(x-x_{i}\right)\left(y-y_{i}\right)+k_{3}{\left(y-y_{i}\right)}^{2})}$$
  being $k_{1}$, $k_{2}$ and $k_{3}$ the coefficients used to take into account the rotation of the function $\beta_{i}$, defined by the relations
  $$\begin{array}{ccc} k_{1}\left(\beta_{i}\right) & = & \frac{cos{\left(\beta_{i}\right)}^{2}}{2\sigma^{2}}+\frac{sin{\left(\beta_{i}\right)}^{2}}{2\sigma_{s}^{2}}\\ k_{2}\left(\beta_{i}\right) & = & \frac{sin(2\beta_{i})}{4\sigma^{2}}-\frac{sin(2\beta_{i})}{4\sigma_{s}^{2}}\\ k_{3}\left(\beta_{i}\right) & = & \frac{sin{\left(\beta_{i}\right)}^{2}}{2\sigma^{2}}+\frac{cos{\left(\beta_{i}\right)}^{2}}{2\sigma_{s}^{2}} \end{array}$$
  where $\sigma_{s}$ is the variance on the left and right ($\beta_{i}\pm \pi /2$ direction) and defines the variance along the $\beta_{i}$ direction ($\sigma_{h}$), or the variance to the rear ($\sigma_{r}$). See [30] for details.
- Social mapping: Space Affordances and Activity Spaces. Let $O_{M}=\left\{o_{1},\ldots ,o_{M}\right\}$ be the set of *M* objects with which humans interact in the environment. The position and type of these objects is information known to the CPS-AAL. Thus, each object $o_{k}\in O_{M}$ stores the interaction space $i_{o_{k}}$ as an attribute, which is associated with the space required to interact with this object and also its pose $p_{o_{k}}=\left(x,y,\theta_{k}\right)$
  (4) $$o_{k}=\left(p_{o_{k}},i_{o_{k}}\right)$$
  Different objects in the environment have different interaction spaces $i_{o_{k}}$. For example, the table for therapies has a smaller space compared to watching TV because the latter interaction can be done from a further distance.

#### 4.3.2. Socially Acceptable Path-Planning Approach

A uniform graph composed of obstacle-free nodes is used to represent the robot’s surroundings. The nodes’ cost varies according to the personal spaces, the activity spaces of the objects, and the social interaction spaces. This paper uses the social mapping described in [32]. Nevertheless, to facilitate the readers’ understanding, the fundamental concepts of this approach are described next:

- Graph-based grid mapping. Space is represented by a graph G(N,E) of *n* nodes, regularly distributed in the environment. Each node $n_{i}$ has two parameters: availability, $a_{n}$, and cost, $c_{n}$. The availability of a node is a Boolean variable whose value is 1 if the space is free, 0 otherwise. The cost, $c_{i}$, indicates the traversal cost of a node, i.e., what it takes for the robot to visit that node (high values of $c_{i}$ indicates that the robot should avoid this path). Initially, all nodes have the same cost of 1 (see [32] for details).
- Social graph-based grid mapping. The space graph G(N,E) includes the social interaction spaces, both for individuals and groups of people, as for objects. The availability $a_{n}$ and the cost $c_{i}$ parameters of each node in these regions are modified accordingly (see [32] for details).

The classical Dijkstra algorithm is used to calculate the optimal path. The optimal path must satisfy two conditions, on the one hand, it must be the shortest path between the origin and destination nodes, and on the other, it must minimize the sum of the costs of the nodes that compose it.

### 4.4. Experimental Results and Discussion

**Figure 12:**
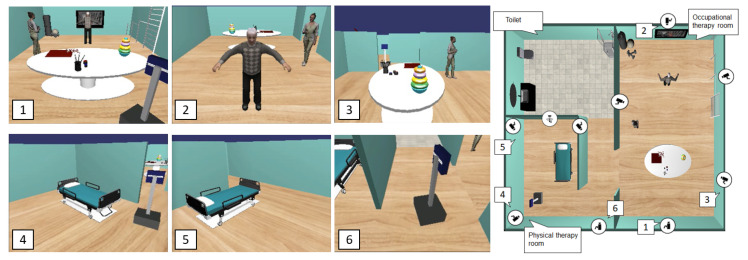
Six RGB images acquired by the caregiving center’s sensor network in different moments. The cameras are tagged to locate them in the environment used for the experiments.

The evaluation of the CPS-AAL for robot’s social navigation in caregiving scenarios requires the correct performance of all architecture agents. Both the detection of people in the scenario and the detection of changes in the objects’ positions are carried out with software agents that use information from RGBD cameras, which means that the visual field of the camera network distributed throughout the environment reaches most of the scenario [22]. Figure 12 shows images acquired by using the camera network deployed in the caregiving center at different instants of time. As the figure shows, there is minimal overlap between cameras, which is needed for the monitoring of people and the SAR during the activities. The CPS-AAL keeps updated information based on the analysis of data provided by the physical world W that has its virtual representation in the digital twin model C.

**Figure 13:**
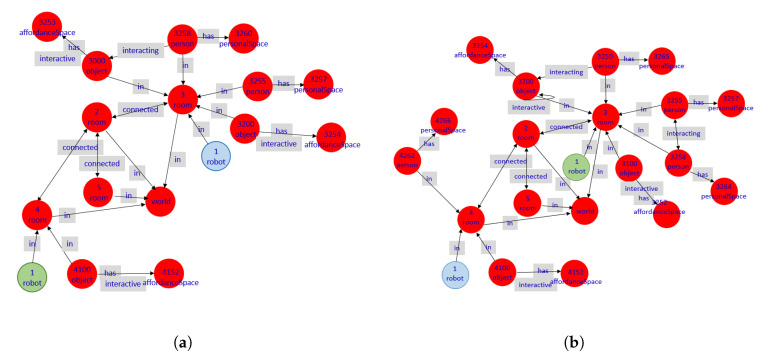
(**a,b**) Graph representation of the digital twin model C corresponding to the physical world W for the two use cases, respectively. The green node labeled as ’robot’ is the SAR location at the beginning of each test. This node, like the SAR in W, moves through the graph. The blue node is the SAR location at the ending of each test.

The digital twin model of the physical world for both scenarios is shown in Figure 13. For both cases, the experimental environment consists of four rooms (i.e., toilet, corridor, occupational, and physical therapy rooms) connected to each other according to the design of the caregiving center. Among the node’s attributes are not only their geometrical dimensions but also environmental parameters, such as temperature, CO${}_{2}$ level, or humidity. Depending on the use case, these four nodes are connected to other nodes associated with people and objects through the in edge. Furthermore, on the one hand, people has personal spaces, and on the other hand, an object in a room has its associated affordance space. If a person is interacting with the interactive object, an edge is also drawn in the graph. This same edge is drawn in the case of two people are interacting with each other.

To validate the social navigation of the SAR in each case of use, a methodology similar to that proposed in [36,37,38] has been carried out, who established a set of metrics to evaluate the navigation of a robot in human environments: (1) average minimum distance to a human during navigation, $d_{min}$; (2) distance traveled, $d_{t}$; (3) navigation time, τ; (4) cumulative heading changes, CHC; and (5) personal space intrusions, Ψ. Nevertheless, a brief description of these metrics is provided:

- Average distance to the closest human during navigation: A measure of the average distance from the robot pose, $x_{r}\left(x,y,\theta \right)$, to the closest human $h_{i}\left(x,y,\theta \right)$ along the robot’s path $P=\left\{x_{r}^{j}\left(x,y,\theta \right)|j=1,2\ldots N\right\}$, being *N* the number of points of the path planned by the agent.
  (5) $$d_{min}=min_{i}\left\{x_{r}{}^{j}\left(x,y\right)-h_{i}\left(x,y\right)\right\}$$
- Distance traveled: length of the path planned by the navigation framework, in meters.
  (6) $$d_{t}=\sum_{j=1}^{j=N-1}x_{r}^{j}\left(x,y\right)-x_{r}^{j+1}\left(x,y\right)$$
- Navigation time: time since the robot starts the navigation, $\tau_{ini}$, until it arrives to the target, $\tau_{end}$.
  (7) $$\tau =\tau_{end}-\tau_{ini}$$
- Cumulative Heading Changes (CHC): a measure to count the cumulative heading changes of the robot during navigation [38]. Angles are normalized between −π and π.
  (8) $$CHC=\frac{1}{N}\sum_{j=1}^{j=N-1}x_{r}^{j}\left(\theta \right)-x_{r}^{j+1}\left(\theta \right)$$
- Personal space intrusions (Psi): In this paper, four different areas are defined: Intimate ($x_{r}^{j}\left(x,y\right)-h_{i}\left(x,y\right)\leq 0.45\;m$); Personal ($0.45\;m\leq x_{r}^{j}\left(x,y\right)-h_{i}\left(x,y\right)\leq 1.2\;m$); Social ($1.2\;m\leq x_{r}^{j}\left(x,y\right)-h_{i}\left(x,y\right)\leq 3.6\;m$); and Public ($x_{r}^{j}\left(x,y\right)-h_{i}\left(x,y\right)\geq 3.6\;m$). This metric measures the percentage of the time spent in each area along the robot’s path as:
  (9) $$Psi=\left\{\frac{1}{N}\sum_{i=1}^{i=N}Fx_{r}^{j}\left(x,y\right)-h_{i}\left(x,y\right)\leq \delta^{k}\right\}$$
  where $\delta^{k}$ defines the distance range for classification (intimate, personal, social and public), and F() is the indicator function.

**Figure 14:**
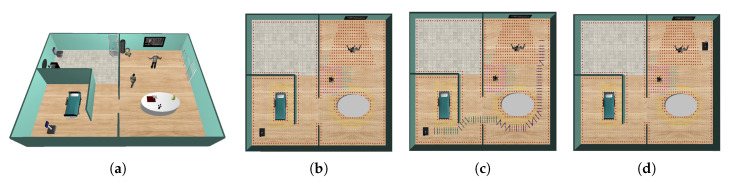
First use case: (**a**) 3D view of the simulated scenario; (**b**) social interaction spaces, both for people (the senior and the caregiver) and the objects in the CPS-AAL; (**c**) path planned by the robot in the CPS-AAL; and (**d**) SAR’s pose at the end of the first use case.

Figure 14 describes the first use case. Figure 14a depicts a 3D view of the scenario with the old adult and the caregiver in the occupational therapy room. Figure 14b illustrates the social interaction spaces of the different agents in the scenario. These social spaces of interaction, defined through models in the digital twin world, modify the free space graph used to plan the path. People add an asymmetric Gaussian-shaped space with different weights depending on whether it is intimate, personal, social, or public space, penalizing the robot’s path through these nodes of the graph [30]. Similarly, objects in the environment generate interaction spaces if the caregiving center’s users are interacting with them. Thus, the route planned by the robot takes into account all these values, and the navigation agent builds a social path to the target pose, in this case, the occupational therapy room to communicate the end of the therapy. The route planned by the robot is shown in Figure 14c. This path avoids crossing close to the people in the room, getting as far away from them as possible, always minimizing the distances traveled. The time it takes for the robot to reach its target increases considerably compared to a classic planner without social behavior, but in return, it does not disturb people while they are performing their therapy (see Table 4). The final robot’s pose is drawn in Figure 14d (Readers can watch the video of this use case at the address: https://youtu.be/hJYLT661TqU). In the video, images acquired from the RGBD camera network are also shown). At this point, the robot is in a position close enough to the older person to be heard, and the interaction can begin. The results of this first use case are shown in Table 4, where metrics for the path planned by a classical Dijkstra’s planner without social behavior is also detailed. First, as is evident, the path planned by the robot without social behavior travels a shorter distance in a shorter time. However, distances to the people $d_{min}^{senior}$ and $d_{min}^{caregiver}$ are very small, which can bother the caregiving center’s users. This same situation can also be observed with the value of Ψ(personal), which indicates that the robot invades this personal space. In the case of social navigation, thanks to CPS-AAL, the robot can plan a socially accepted path, which allows it to reach the target position without bothering anyone, as shown the values of Ψ in Table 4 equal to zero in all cases, except for the public area.

**Figure 15:**
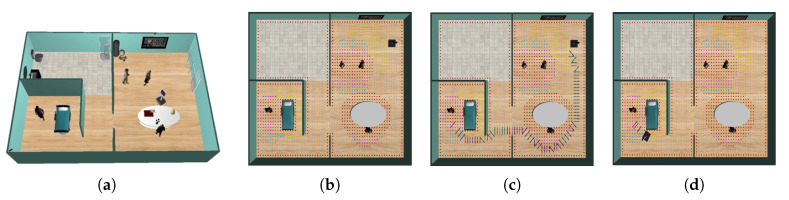
Second use case: (**a**) 3D view of the simulated scenario; (**b**) social interaction spaces, both for people (the seniors and the caregiver) and the objects in the CPS-AAL; (**c**) path planned by the robot in the CPS-AAL; and (**d**) SAR’s final pose at the end of the second use case.

Figure 15 describes the second use case. In this scenario, two people will interact with each other, and the robot should avoid passing near them to move to the physical therapy room (see Figure 15a). Social spaces of interaction are shown in Figure 15b. As in the previous use case, the models of the digital twin world, the affordance spaces for objects and the asymmetric Gaussian spaces for people, modify the free space graph. The planned route is shown in Figure 15c. In this case, the robot searches for the optimal path respecting the social norms until it reaches the final position, where the interaction with the old adult begins (Figure 15d) (A video of this second use case can be found in: https://youtu.be/Npb-kfNRLpo).

Table 5 shows the set of metrics obtained after performing the social navigation framework within the proposed CPS-AAL. These metrics are compared with a classical Dijkstra’s path-planning algorithm without social behavior. As in the first test, the results show how the robot’s social behavior needs a longer path, and therefore it also needs more time to perform it. However, this social behavior prevents the robot from navigating near people, as the values of Ψ and $d_{min}$ show.

As a summary of the experiments, it can be concluded that the SAR presents notable advantages in social navigation behavior, avoiding navigating near people (caregivers or older people) or invading areas where people interact with objects during therapy. All this would be much more complicated without a system that works in a coordinated way and integrates the physical world with specific models and agents that support the whole system. In the solution presented in this work, the cyber-world, built from the digital twin model with a shared working memory, the DSR, and the CORTEX architecture, facilitates the coordinated work of the agents and reduces the complexity of the problems. Finally, the metrics used in this work promote the comparison of the proposed approach with other similar works in the literature. The social navigation framework can be effortlessly adapted to changes and modifications, due to the essential feature in the complete system is the integration of the two worlds, the physical and the cyber-world, and the architecture presented here meets the desired criteria, including being modular and easily scalable.

## 5. Conclusions

The deployment of digital technologies in caregiving centers to make future decisions that can be useful for assisting elderly and caregivers is becoming a reality thanks to the advance of technologies such as the Internet of things, data science, or cloud computing. The future of these centers is to endow their facilities with a sufficient set of devices—physical world—to provide users with tools to increase their safety, optimize the results of physical and cognitive therapies, as well as to provide solutions that provide elderly with a more independent and better quality of life. In this context, the use of Cyber-Physical Systems is conceived as a powerful tool that integrates most of the above technologies to create an ideal framework to achieve these objectives. These CPSs have made the leap from the industry to other sectors, such as agriculture, medicine, transport, and in recent years, although at plodding speed, to hospitals or nurse homes.

This paper describes, following a similar nomenclature to other papers, a specific CPS for caregiving centers named CPS-AAL, detailing each of the components and agents that form the complete system. As a novelty, the proposal includes people and a socially assistive robot as integral parts of the CPS. This SAR has, among others, essential skills to navigate and interact with users. The CPS-AAL presented in this work uses a digital twin-world model with all the information acquired by physical devices and shared by the rest of the agents involved. The basis of this cyber-world is the CORTEX cognitive architecture, a set of software agents that interact with the shared information.

The CPS description is not complete if it is not validated against a use case that requires the interaction of the different components and agents. For this reason, this work presents two use cases where the CPS-AAL is used in the problem of socially accepted navigation. For this purpose, data collected by the physical world are used in the digital twin model for the detection and tracking people in the caregiving center, for the detection of objects and possible interactions between people and these objects, as well as for planning a robot’s path that does not disturb people. This navigation framework within the CPS-AAL, impossible to carry out successfully without an architecture that includes different devices deployed in the environment, is described and validated in this work. As a summary of the experiments, it can be concluded that the robot presents notable advantages in social navigation behavior, avoiding situations that are not socially accepted, such as invading the space of interaction between an object and a person or between people. The metrics used in this paper facilitates the comparison of the proposed approach with other similar state-of-art works.

The possibilities of extending this work are diverse. One interesting direction is to extend the use case to cover other essential tasks in a caregiving center, such as monitoring the elderly to detect falls, observe the intake of medication, or automatic performing and monitoring occupational therapies. Another line of research is to extend CORTEX, and by extension, the digital twin model, with more modeling power and with predictive capabilities. The self and world representation maintained in the working memory can be augmented with a temporal dimension into the future and the past. With the inclusion of specialized simulators, such us physics or human activity simulators, the system could anticipate the outcome of potential actions and exhibit a more proactive and socially aware behavior with humans. 

## Figures and Tables

**Table 1:** Devices list of the CPS-AAL proposed.

Category				Robotic Application	
Type	Data	Purpose	Format	Assistive	Social
Environmental	Temperature	Measure room temperature	Time series	x	
	Humidity	Measure room humidity	Time series	x	
	CO${}_{2}$	Measure room CO${}_{2}$ ppm	Time series	x	
	People presence	Motion detection	Categorical	x	x
Personal	RGB/RGBD cameras	Monitoring and tracking, daily activity detection, …	Multimedia	x	x
	Microphone	Voice detection, HRI	Audio	x	x
	Speakers	Alerts and instructions, HRI	Audio	x	x
	Tactile TV/monitor	Visual information, HRI	Multimedia	x	x
	sonar/laser	Robot navigation	Time series	x	

**Table 2:** Use case 1: The robot acts as an assistant that warns the user that the therapy is over.

Actor	Action
**Caregiver**	The caregiver keeps the therapy schedule updated on the center’s calendar
**Senior**	The user performs his scheduled activity in the occupational therapy room
**Physical World**	
RGBD cameras	RGBD cameras collect the data of the caregiving center useful for navigation
Microphones/speakers	The microphones/speakers of the robot and the environment are used in the phase of interaction with the users
Communication	This data is sent via Ethernet
**Digital Twin Model**	
Object detection agent	The agent estimates the position of the objects, and if there have been changes, updates the DSR
Person detection agent	The agent estimates the position of the users and updates the DSR
Caregiving center management agent	When the time of the end of the activity is reached, the module triggers an alert service to the robot.
SAR	Once the reminder is received, the robot launches its plan: to reach the occupational therapy room
Social navigation agent	The agent plans a socially acceptable path and navigates it to its goal.
HRI agent	The agent interacts with users to warn them of the end of the activity
**Senior**	The user leaves the room
**Physical World**	Physical devices corroborate that users leave the room

**Table 3:** Use case 2: The robot acts as a virtual physical therapist that navigates to the user and proposes a physical activity.

Actor	Action
**Caregiver**	The caregiver keeps the therapy schedule updated on the center’s calendar
**Senior**	The user waits in the physical therapy room
**Physical World**	
RGBD cameras	RGBD cameras collect the data of the caregiving center useful for navigation
Microphones/speakers	The microphones/speakers of the robot and the environment are used in the phase of interaction with the users
Communication	This data is sent via Ethernet
**Digital Twin Model**	
Object detection agent	The agent estimates the position of the objects, and if there have been changes, updates the DSR
Person detection agent	The agent estimates the position of the users and updates the DSR.
Caregiving center management agent	When the time of the end of the activity is reached, the module triggers an alert service to the robot
SAR	Once the reminder is received, the robot launches its plan: to reach the physical therapy room
Social navigation agent	The agent plans a socially acceptable path and navigates it to its goal
HRI agent	The agent interacts with users to warn them of the start of the activity
Physical therapy agent	The agent interacts with users and launch the therapy
**Senior**	The user performs the physical activity, interacting with the touch screen and by voice message
**Physical World**	The physical devices corroborate that the users correctly perform the activity proposed by the robot

**Table 4:** Results of the social navigation framework for the social robot in the first use case. A detailed description of the metrics and a brief discussion can be found in the text.

	Social Path-Planning	Classical Dijkstra’s Path-Planning
Parameter	Value	Value
*$d_{t}$ (m)*	15.01	12.21
*τ (s)*	46.64	36.21
*CHC*	7.42 (1.27)	8.23
*$d_{min}^{caregiver}$(m)*	2.88	1.23
*$d_{min}^{senior}$ (m)*	2.14	1.13
Ψ *(Intimate) (%)*	0.0	0.0
Ψ *(Personal)(%)*	0.0	11
Ψ *(Social)(%)*	0.0	8
Ψ *(Public)(%)*	100.0	81.0

**Table 5:** Results of the social navigation framework for the social robot in the first use case. A detailed description of the metrics and a brief discussion can be found in the text.

	Social Path-Planning	Classical Dijkstra’s Path-Planning
Parameter	Value	Value
*$d_{t}$ (m)*	16.27	12.54
*τ (s)*	72.68	61.22
*CHC*	1.52 (0.6)	2.32
*$d_{min}^{senior1}$ (m)*	4.13	3.35
*$d_{min}^{caregiver}$ (m)*	2.70	0.85
*$d_{min}^{senior2}$ (m)*	1.125	3.45
*$d_{min}^{senior3}$ (m)*	1.318	1.318
Ψ *(Intimate) (%)*	0.0	2.23
Ψ *(Personal)(%)*	1.31	6.36
Ψ *(Social)(%)*	8.23	10.01
Ψ *(Public)(%)*	90.46	81.04

## Abbreviations

The following abbreviations are used in this manuscript:

AAL	Ambient Assisted Living
CPS	Cyber-Physical System
DSR	Deep State Representation
IoT	Internet of Things
SAR	Socially Assistive Robot
